# Recurrent Rhinophyma: A Case of Triple Relapse and Therapeutic Challenges

**DOI:** 10.1002/ccr3.72690

**Published:** 2026-05-12

**Authors:** Bilal Aslam, Muhammad Abbas, Syed Muhammad Tayyab, Khalil El Abdi, Fazeela Bibi, Hania Imran, Samreen Najeeb, Ahmad Sanan, Said Hamid Sadat

**Affiliations:** ^1^ University of Lahore Lahore Pakistan; ^2^ HOD Plastic Surgery University of Lahore Lahore Pakistan; ^3^ Faculty of Medicine and Pharmacy of Rabat Mohammed V University Rabat Morocco; ^4^ Jinnah Medical and Dental College Karachi Pakistan; ^5^ Wah Medical College Wah Pakistan; ^6^ Women Medical & Dental College Abbottabad Pakistan; ^7^ Khyber Medical College Peshawar Pakistan; ^8^ Kabul University of Medical Science Abu Ali Ibn Sina Kabul Afghanistan

**Keywords:** case report, nasal reconstruction, phymatous rosacea, recurrence, rhinophyma, surgical excision, tangential excision

## Abstract

Rhinophyma recurrence after surgical excision poses a significant therapeutic challenge, often stemming from the incomplete removal of deep hyperplastic pilosebaceous units. We present the case of a 66‐year‐old male with a third occurrence of rhinophyma after two prior failed cold‐steel excisions. The definitive intervention consisted of a modified deep tangential excision, removing all rhinophymatous tissue to a deep dermal plane that approached, but did not violate, the nasal perichondrium, thereby eradicating the pathologic glandular nidus while preserving the deepest adnexal remnants essential for re‐epithelialization. The wound was primarily managed by secondary intention healing, guided by selective, tension‐free suture re‐approximation of key wound edges to optimize contour. Cold‐steel excision was specifically chosen to ensure a specimen free of thermal artifact for definitive histopathology, a critical step in a recurrent case, which ultimately confirmed marked sebaceous gland hypertrophy and excluded malignancy. The postoperative course was uneventful, and at an 18‐month follow‐up, the patient demonstrated a stable, successfully restored nasal contour with no signs of recurrence. This case demonstrates that for severe, recurrent rhinophyma, a surgical strategy that balances thorough debulking of pathologic tissue with meticulous preservation of deep adnexal structures is critical for achieving a durable, long‐term outcome.

## Key Clinical Message

For rare cases of multi‐recurrent rhinophyma, superficial debulking is insufficient and leads to relapse. A durable outcome requires a modified deep tangential excision to eradicate the nidus of hyperplastic glandular tissue, balancing radical removal with the preservation of the deepest adnexal structures necessary for healing by secondary intention.

## 1 Introduction

Rhinophyma represents a severe, disfiguring manifestation of phymatous rosacea, characterized by progressive glandular hypertrophy and fibroplasia of the nasal soft tissues [1, 2]. Standard surgical treatments, including tangential cold‐steel excision, dermabrasion, and ablative carbon dioxide (CO_2_) laser, are typically effective, and recurrence after an adequate primary intervention is uncommon.

Relapse, when it occurs, is frequently attributed to the incomplete extirpation of deep‐seated pilosebaceous units that serve as a nidus for regrowth. However, the literature offers limited guidance on optimal strategies for managing patients after multiple surgical failures [3]. We present a case of rhinophyma recurring after two prior surgical treatments to delineate the meticulous surgical principles essential for achieving a durable outcome in such recalcitrant scenarios.

## 2 Case History

**FIGURE 1:**
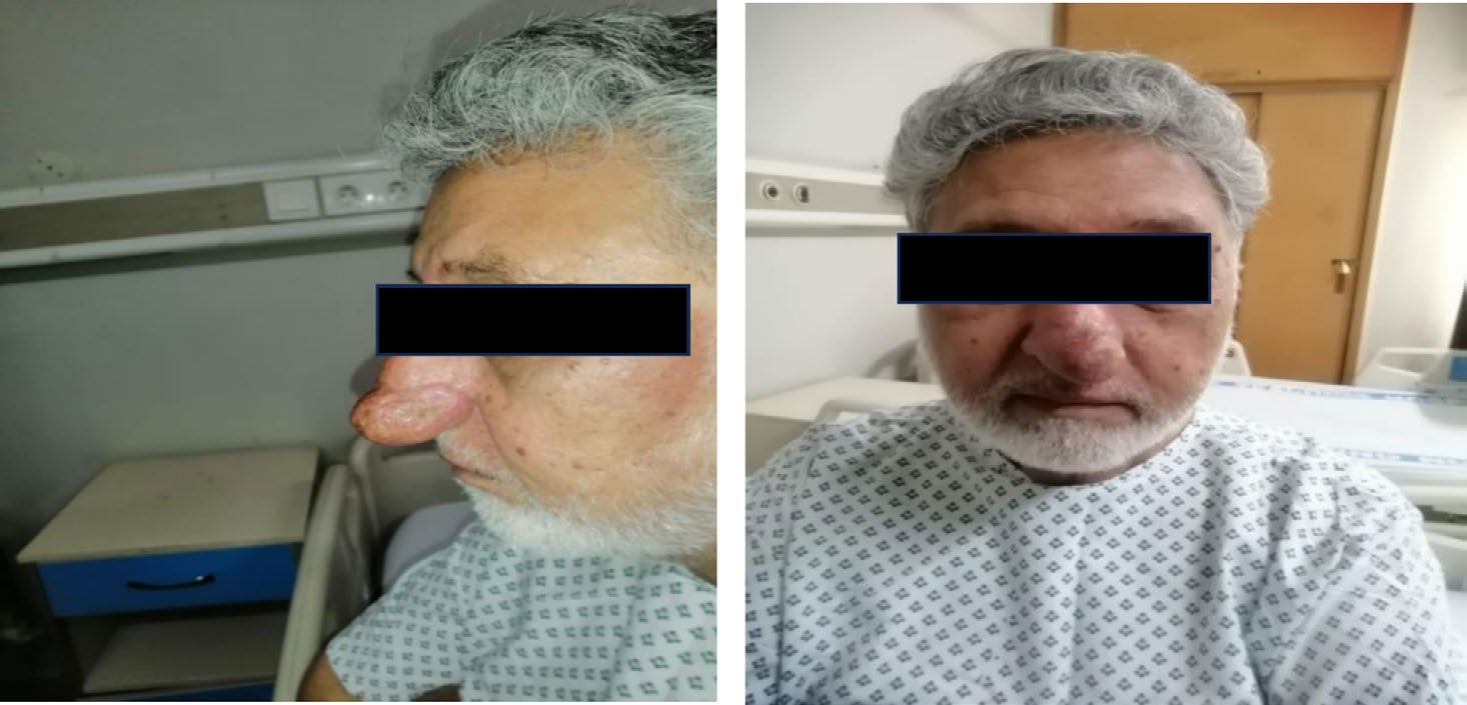
Preoperative clinical photograph of a patient with rhinophyma. The image displays significant, disfiguring soft tissue hypertrophy and swelling predominantly affecting the left side of the nose 1 2. This presentation is characteristic of advanced phymatous rosacea, marked by sebaceous gland hyperplasia and fibrosis.

A 66‐year‐old male presented with a progressively enlarging and disfiguring nasal mass (Figure 1). His clinical history was notable for a third occurrence of rhinophyma, for which he had undergone two separate surgical excisions four years prior using a cold‐steel tangential debulking technique. Despite these interventions, the patient experienced significant regrowth of the nasal tissue, suggesting the prior procedures had been of insufficient depth to remove the hyperplastic glands completely. He denied associated pain or ulceration, and his medical and family histories were non‐contributory.

Physical examination revealed a prominent, asymmetric, and lobulated deformity of the external nose, predominantly affecting the left nasal ala. The overlying skin exhibited pronounced cutaneous thickening, diffuse erythema, and patulous follicular orifices consistent with extensive sebaceous hyperplasia. Regional lymphadenopathy was absent.

## 3 Diagnostic Investigations

A preoperative magnetic resonance imaging (MRI) scan of the face was performed to delineate the extent of the soft tissue hypertrophy and to exclude infiltration into the underlying cartilage or bone, given the history of multiple recurrences. The MRI demonstrated heterogeneous enhancement of the enlarged external nasal tissues without evidence of invasion into the nasal framework.

**FIGURE 2:**
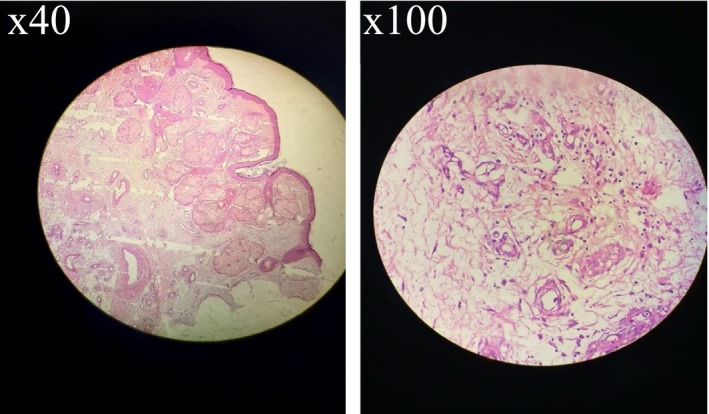
Microscopic view of rhinophyma tissue, showing hallmark histopathological changes. As a manifestation of severe phymatous rosacea, the tissue exhibits prominent hyperplasia and hypertrophy of sebaceous glands, a primary contributor to the clinical enlargement of the nose. The slide also demonstrates significant dermal fibrosis, characterized by thickened and sclerotic collagen bundles, alongside dilated hair follicles. Key vascular abnormalities are visible as telangiectasias. A notable feature is the chronic inflammatory infiltrate, rich in lymphocytes, surrounding the blood vessels and hair follicles, which underscores the inflammatory nature of the disease.

**FIGURE 3:**
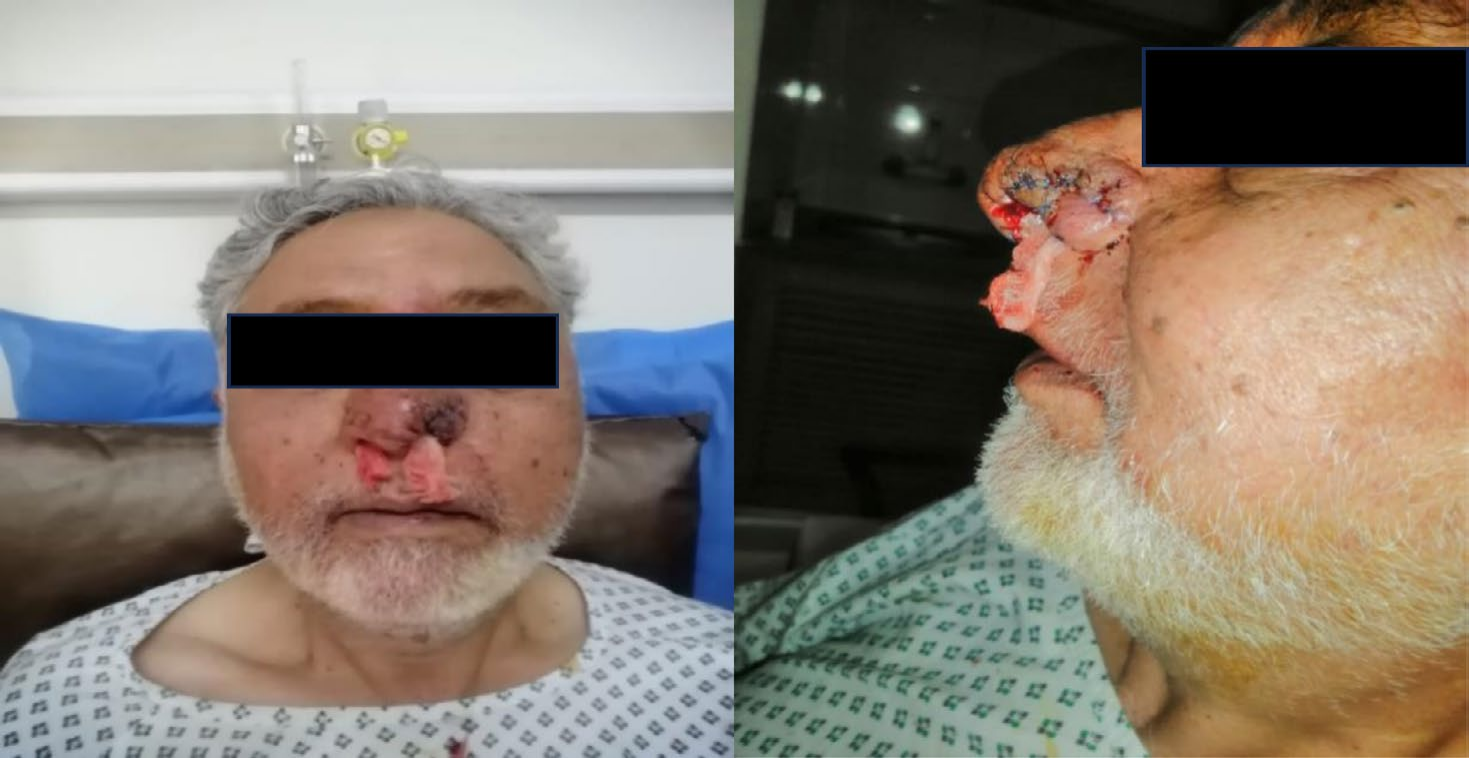
Immediate postoperative result following surgical intervention for rhinophyma. The image shows the nasal contour after surgical excision and debulking of the hypertrophic tissue, with sutures in place. This procedure aims to restore a more conventional nasal shape and function.

Definitive diagnosis was established through histopathological analysis of the excised surgical specimen (Figures 2 and 3). Microscopic examination confirmed the clinical impression, revealing stratified squamous epithelium with marked acanthosis and pronounced sebaceous gland hypertrophy. The dermis exhibited a mild perifollicular inflammatory infiltrate rich in plasma cells. Crucially, this step is imperative in all cases of rhinophyma to formally exclude differential diagnoses; no evidence of malignancy, such as basal cell carcinoma, was detected in the specimen.

All preoperative laboratory investigations were within normal limits.

## 4 Treatment, Outcomes, and Follow‐Up

**FIGURE 4:**
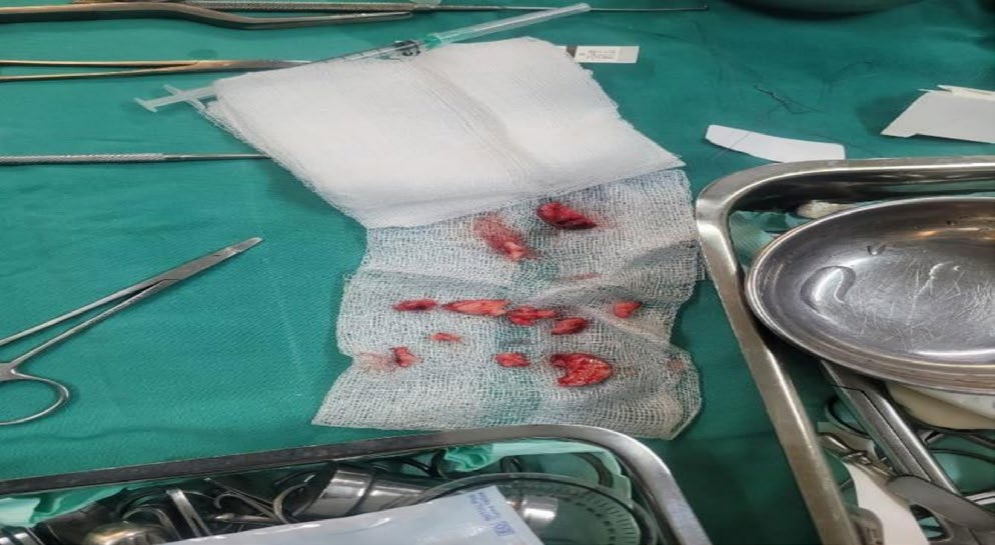
Gross specimen of the excised nasal tissue prepared for analysis. The tissue fragments were sent for histopathological examination to confirm the diagnosis of rhinophyma and to rule out any underlying malignancy, which is a crucial step in management.

The surgical intervention was performed under general anesthesia, predicated on the principle of complete tangential excision of all hypertrophic tissue (Figure 4). Using a sharp scalpel, the rhinophymatous mass was meticulously debulked in a deep tangential plane within the reticular dermis. The excision continued until the bulk of hyperplastic glands was removed, creating a plane that approached, but did not violate, the underlying nasal perichondrium, thereby preserving the deepest adnexal remnants for re‐epithelialization. This approach was designed to remove the bulk of the hyperplastic sebaceous glands while carefully preserving the deepest adnexal structures necessary for re‐epithelialization. Hemostasis was achieved with pinpoint electrocautery, which was also used for final, detailed contouring of the nasal subunits.

Following complete excision, the majority of the surgical site was dressed with a non‐adherent petrolatum gauze and left to heal by secondary intention. To guide the final aesthetic result, limited undermining of adjacent nasal skin allowed for the partial, tension‐free re‐approximation of select wound edges with 4‐0 nylon sutures, aiding in the achievement of an optimal final contour.

The postoperative course was uneventful, with no signs of infection, hypertrophic scarring, or functional compromise. The patient reported a high degree of satisfaction with both the functional and cosmetic outcomes. At an 18‐month follow‐up, clinical examination confirmed excellent wound healing, a successfully restored and stable nasal contour, and no evidence of recurrence or contracture.

## 5 Discussion

The management of advanced or recurrent rhinophyma poses a formidable therapeutic challenge, centered on achieving complete and lasting tissue removal while restoring a normal nasal contour. A critical distinction must be made between early regrowth from incomplete treatment and true, late recurrence [3, 4]. Contrary to the impression given by challenging cases like this one, recurrence after an adequate primary surgical intervention is uncommon. For instance, a recent long‐term study of 152 patients treated with fully ablative CO_2_ laser reported a low recurrence rate of only 4%, occurring primarily in older male patients with the most advanced disease [5]. This patient's history of a third occurrence following prior cold‐steel excisions strongly suggests the initial procedures were of insufficient depth to completely eradicate the hyperplastic pilosebaceous units.

The surgical principle for a definitive cure requires a meticulous balance: the intervention must extend deep enough to ablate the pathologic, hypertrophic adnexal structures that serve as a nidus for regrowth, while simultaneously preserving the deepest basal glandular remnants at the dermo‐subcutaneous junction, which are essential for re‐epithelialization and healing by secondary intention [3]. Excision down to bare cartilage would eliminate this regenerative capacity, often necessitating skin grafting and increasing the risk of scarring and textural abnormalities [6]. Our modified deep tangential technique was designed to achieve this balance.

While ablative CO_2_ laser resurfacing is widely considered a gold standard for its precision and superior hemostatic properties, cold‐steel excision was the optimal choice for this patient. The primary rationale was to ensure the availability of a large, high‐quality specimen for histopathological analysis without thermal artifact—a critical step in recurrent cases to definitively exclude occult malignancy. A secondary, practical consideration was its universal availability and cost‐effectiveness compared to capital‐intensive laser systems.

This case also highlights an important nuance regarding hemostasis. While cold‐steel excision provides a specimen free of thermal artifact, hemostasis and final contouring of the wound bed were achieved with pinpoint electrocautery. This represents a necessary clinical trade‐off. Judicious use of cautery is essential to control bleeding, but it inevitably introduces some degree of thermal energy to the remaining deep dermal and adnexal structures. The successful outcome suggests that careful application can preserve sufficient viable tissue for robust re‐epithelialization.

Finally, this case underscores that histopathological examination is imperative in all excisions of rhinophyma. Although a definitive causal link between rhinophyma and basal cell carcinoma (BCC) remains unsubstantiated, the possibility of a collision tumor or a malignancy mimicking phymatous changes necessitates a thorough microscopic analysis to ensure an accurate diagnosis and appropriate management.

## 6 Conclusion

This case of a triple‐relapse rhinophyma underscores the profound therapeutic challenge posed by recurrence and reinforces the principle that successful long‐term outcomes are contingent upon the complete and meticulous ablation of deep‐seated hyperplastic sebaceous glands. Superficial debulking is insufficient and predisposes to relapse. Our experience demonstrates that for recalcitrant cases, a modified deep tangential excision—aggressively removing pathologic tissue down to a deep dermal plane that preserves the deepest adnexal structures for re‐epithelialization—offers a definitive solution.

This technique balances the need for radical excision with the physiological requirements for healing by secondary intention, thereby avoiding the morbidity of full‐thickness resection and skin grafting. The 18‐month recurrence‐free outcome in this patient validates this approach. Ultimately, this report serves as a critical reminder that while advanced technologies like the CO_2_ laser enhance surgical precision, the fundamental determinant of success in managing severe, recurrent rhinophyma lies in the uncompromising radicality of the excision to ensure no pathological tissue remains. This surgical principle, validated by histopathological analysis, is the cornerstone of preventing future relapse and achieving lasting patient satisfaction.

## Author Contributions

**Bilal Aslam:** conceptualization, data curation, validation, visualization, writing – original draft, writing – review and editing. **Muhammad Abbas:** conceptualization, data curation, validation, visualization, writing – original draft, writing – review and editing. **Syed Muhammad Tayyab:** formal analysis, funding acquisition, investigation, methodology, project administration, visualization, writing – original draft, writing – review and editing. **Khalil El Abdi:** conceptualization, data curation, formal analysis, investigation, methodology, project administration, resources, validation, visualization, writing – original draft, writing – review and editing. **Fazeela Bibi:** conceptualization, data curation, formal analysis, funding acquisition, writing – original draft, writing – review and editing. **Hania Imran:** investigation, methodology, project administration, writing – original draft, writing – review and editing. **Samreen Najeeb:** conceptualization, data curation, validation, visualization, writing – original draft, writing – review and editing. **Ahmad Sanan:** conceptualization, supervision, validation, visualization, writing – original draft, writing – review and editing. **Said Hamid Sadat:** conceptualization, investigation, methodology, project administration, writing – original draft, writing – review and editing.

## Funding

The authors have nothing to report.

## Ethics Statement

Written informed consent was obtained from the patient to publish this case report.

## Conflicts of Interest

The authors declare no conflicts of interest.

## Data Availability

No additional data are available beyond those contained in this article and its Supporting Information files.
